# Spatial distribution and determinants of health loss from Kashin-Beck disease in Bin County, Shaanxi Province, China

**DOI:** 10.1186/s12889-021-10407-6

**Published:** 2021-02-19

**Authors:** Jing Wang, Xiaoya Wang, Hairong Li, Linsheng Yang, Yingchun Li, Chang Kong

**Affiliations:** 1 Key Laboratory for Geographical Process Analysis & Simulation, Research Institute of Sustainable Development, Central China Normal University, Wuhan, 430079 China; 2 Key Laboratory of Land Surface Pattern and Simulation, Institute of Geographical Sciences and Natural Resources Research, Chinese Academy of Sciences, Beijing, 100101 China; 3 College of Resources and Environment, University of Chinese Academy of Sciences, Beijing, 100049 People’s Republic of China; 4 Binxian Center for Disease Prevention and Control, Binxian, 713500 China

**Keywords:** Kashin-Beck disease (KBD), Health loss, Spatial autocorrelation, Selenium

## Abstract

**Background:**

Kashin-Beck disease (KBD) is one of the major endemic diseases in China, which severely impacts the physical health and life quality of people. A better understanding of the spatial distribution of the health loss from KBD and its influencing factors will help to identify areas and populations at high risk so as to plan for targeted interventions.

**Methods:**

The data of patients with KBD at village-level were collected to estimate and analyze the spatial pattern of health loss from KBD in Bin County, Shaanxi Province. The years lived with disability (YLDs) index was applied as a measure of health loss from KBD. Spatial autocorrelation methodologies, including Global Moran’s I and Local Moran’s I, were used to describe and map spatial clusters of the health loss. In addition, basic individual information and environmental samples were collected to explore natural and social determinants of the health loss from KBD.

**Results:**

The estimation of YLDs showed that patients with KBD of grade II and patients over 50 years old contributed most to the health loss of KBD in Bin County. No significant difference was observed between two genders. The spatial patterns of YLDs and YLD rate of KBD were clustered significantly at both global and local scales. Villages in the southwestern and eastern regions revealed higher health loss, while those in the northern regions exhibited lower health loss. This clustering was found to be significantly related to organically bound Se in soil and poverty rate of KBD patients.

**Conclusions:**

Our results suggest that future treatment and prevention of KBD should focus on endemic areas with high organically bound Se in soil and poor economic conditions. The findings can also provide important information for further exploration of the etiology of KBD.

**Supplementary Information:**

The online version contains supplementary material available at 10.1186/s12889-021-10407-6.

## Background

Kashin-Beck disease (KBD) is a chronic, endemic, deformative osteoarthropathy, which is known for the formation of multi-joint hyperplasia bone changes [1]. The disease usually starts in childhood and attacks the growth plate of articular cartilage [2]. Patients with mild KBD have symptoms of joint thickening, occasional mild muscle atrophy, and often accompanied by pain; while those with serious KBD manifest developmental disorders, short limbs and malformation, loss of labor capacity, and confined self-care ability [3]. KBD has been discovered since the sixteenth century, and it is distributed diagonally from northeastern China to Tibet in the southwest, with additional endemic regions in Siberia and North Korea [4]. In China, KBD has been effectively controlled and even eliminated in most affected areas, but the number of existing patients is huge. According to the 2018 health statistics issued by the Chinese Ministry of Health [5], there were currently 177,018 individuals affected by KBD in 379 counties of 13 provinces or autonomous regions, and the largest number was found in Shaanxi Province (60,157 individuals), accounting for 34.0% of the total existing patients with KBD. The life quality of these existing patients still needs to be taken seriously.

Although the prevalence of KBD has reached the level of control in Shaanxi Province [6], the harm of KBD to human health still exists. For a long time, the severity of KBD has been evaluated by the prevalence rate or X-ray positive rate of children, which can only count the number of cases and cannot value the harm of the non-fatal disability caused by different degrees of KBD. By contrast, the disability adjusted life year (DALY) is a time-based metric, proposed by the World Bank and World Health Organization (WHO) to estimate the burden of disease [7]. DALY is the sum of years of life lost due to premature mortality (YLLs) and years lived with disability (YLDs), which quantify the health loss of both fatal and non-fatal consequences [8]. As far as the non-fatal KBD is concerned, its loss of healthy life years is mainly caused by YLDs, and there is no YLLs. DALYs have been widely used to reflect health gaps of diseases among age-sex groups and regions [9–12]. However, to the best of our knowledge, there have been no relevant research on KBD. Given its serious impacts on the health and life quality of patients, an estimation of its health loss is quite necessary. Furthermore, a better understanding of the spatial distribution of health loss caused by KBD will be helpful to identify areas and populations at high risk so as to plan for targeted interventions.

The health loss of KBD is directly related to the severity of KBD. Selenium (Se) deficiency is acknowledged as an important environmental risk factor for the onset of KBD [3, 13, 14]. A great number of studies have reported the close link between the prevalence of KBD and low-Se environment [15–17]. Nevertheless, little is known about the relationship between the health loss of KBD and Se in the environment. The exploration of their relationships may provide a new perspective for the etiologic research of KBD. In addition, socio-economic factors such as educational attainment [18, 19] and family income [20, 21] can also affect the health loss from diseases in varying degrees. Although a few studies have investigated the key factors (i.e., age, educational attainment, severity of KBD, economic level, etc.) influencing the health-related quality of life of adult patients with KBD in Shaanxi Province [22, 23], the knowledge of social determinants contributing to the health loss of KBD is still limited.

Bin County in Shaanxi Province, as one of the most serious KBD endemic areas, was chosen as the study area. In this study, basic conditions of KBD areas and patients in Bin County were investigated at the village-scale. Corresponding environmental samples including cultivated topsoil and wheat were collected to analyze concentrations of total Se and soil Se speciation. The main objectives were to 1) quantify the health loss of KBD based on the YLDs metric; 2) analyze the spatial distribution of YLDs and YLD rate; and 3) explore factors influencing the health loss of KBD from natural and social environment. The results will provide theoretical basis for assisting public health officers to optimize the allocation of health resources and to prevent and control endemic diseases.

## Methods

### Study area

**Fig. 1:**
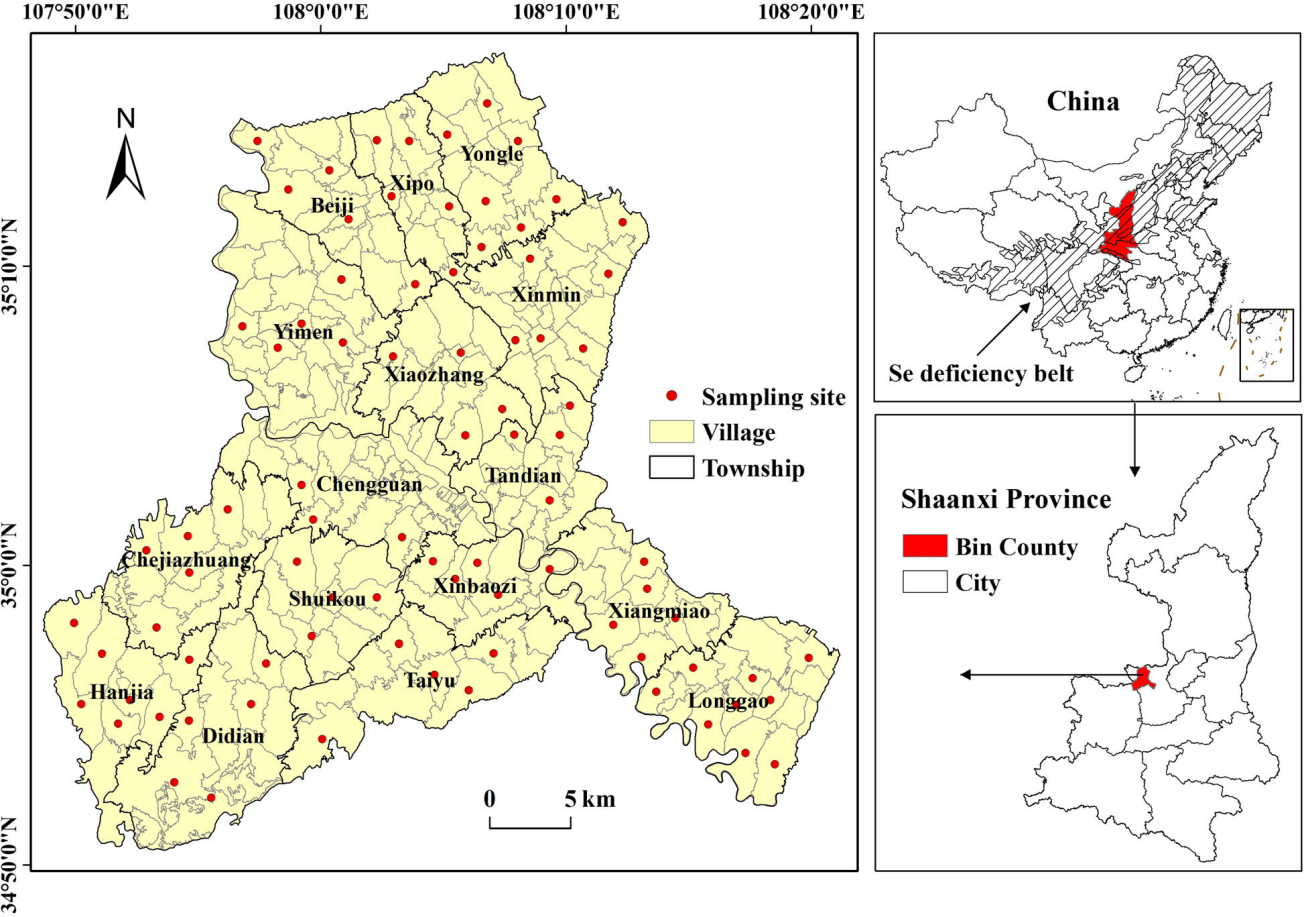
Location of Bin County and distribution of sampling sites

Bin County is located in the central and western part of Shaanxi Province and the middle of the natural Se deficiency belt in China (Fig. 1). It is a national surveillance site for KBD. KBD in Bin County used to be very serious, the prevalence rate of which had been the highest in China from 1992 to 1995 [24]. With the measures of selenium supplement, water source changes and economic development, the prevalence of KBD was under control basically. In 2007, the X-ray detection rate in Bin County was only 0.43%, which had reached the national standard [6]. There have been no new cases discovered in children in recent years, but KBD in adults is still very serious. The location of Bin County is in the hilly and gully area of the southwest of the Loess Plateau, where the terrain tilts from southwest to northeast. The altitude is 791–1482 m. Jing River runs through Bin County from the northwest to the southeast, dividing it into the geomorphic pattern of hilly and gully areas in the northeast and southwest and valley plain areas in the middle. The soil types in Bin County are mainly loessial soil, dark loessial soil and red soil. The climate of the area has the typical warm temperate semi-arid continental monsoon characteristics, with the average annual temperature of 9.7 °C and average annual rainfall of 579 mm [25].

### Data sources

The epidemiological data used for the calculation of YLDs were obtained from the general survey of KBD in Bin County, which was carried out by the Binxian Center for Disease Prevention and Control in 2018. The survey aimed to investigate the prevalence of KBD, including the number of existing patients and the degree of KBD. The basic demographic and socioeconomic information (such as age, gender, education level, occupation, place of residence, medical history, etc.) for each patient with KBD were also recorded. A total of 296,770 people (17.3% were children aged under 12 years) from 244 villages in 16 townships were examined in the survey, whose gender ratio was 1.12:1 (male to female). The data were collected through person-to-person interviews and clinical examination. The interview guide for patients with KBD can be found in Additional file 1.

### Sampling and analyses

Se contents in the environment in Bin County were analyzed to explore factors influencing the health loss of KBD. According to the distribution of KBD affected villages and cultivated land in Bin County, 84 villages were selected based on a 3 × 4 km regular grid to collect cultivated topsoil samples (0–20 cm). The location of sampling sites is shown in Fig. 1. Three sub-samples were taken from the farmland with relatively uniform distance in each village, which were then mixed together to create a composite sample. At each sampling site, wheat samples were randomly collected from a household. Eventually, 90 cultivated topsoil samples and 75 wheat samples were obtained in the study area. The determination of total Se referred to national standards on the determination of Se in foods (GB/T 5009.93–2003) and soil (NY/T 1104–2006). Furthermore, three different fractions of soil Se (i.e. water-soluble, exchangeable, and alkali-soluble organically bound) were extracted successively. The details of the method were represented in our previously published paper [26]. Se in soil and wheat samples were determined by hydrogen generation-atomic fluorescence spectrometry (AFS-9780, HaiGuang Instruments, Beijing, China), the detection limit of which is 0.02 ng/ml and RSD is less than 1.0%. Reagent blanks, duplicated samples, and national standard reference materials (GBW10011 for wheat and GBW07410 for Tibetan soil) were used for analytical quality control.

### Calculation of YLDs and YLD rate

The YLDs can be calculated from either an incidence perspective or a prevalence perspective [9]. The former is the product of incidence, disability weights and average duration of disease; the latter is the product of prevalence of disease and disability weights, which is convenient for comparison with the recent GBD studies [27]. In this study, the prevalence-based YLDs were calculated for the analysis. Discounting and age weighting were not applied. The basic formula is as follows:

$$ YLD=P\times DW $$

where *P* is the number of prevalent cases of KBD; *DW* is the disability weight of different degrees of KBD. DW is usually estimated based on evaluation scales [9, 28] or referenced from the results of Global Burden of Disease (GBD) studies [12, 29]. However, no paper has previously reported disability weights of KBD. Considering that KBD has many similarities with rheumatoid arthritis in clinical manifestations [30], disability weights of rheumatoid arthritis in the latest GBD 2017 study were directly adopted in this study. Thus, disability weights of KBD in different grades were assigned as 0.117 (grade I), 0.317 (grade II) and 0.581 (grade III) in the light of the sequela of rheumatoid arthritis [31].

The YLD rate (YLDs per 1000 population) is calculated from the YLDs in different cohorts divided by the total target population and then multiplied by 1000.

### Spatial autocorrelation analysis

Clustering in the health loss from KBD was analyzed using both global and local spatial autocorrelation statistics. First, the global Moran’s I test statistic was computed to test the null hypothesis of no significant clustering of YLDs and YLD rate in the entire study region [32]. The values of Moran’s I range from − 1 (dispersed) to 1 (clustered). The threshold value of Moran’s I index is 0, indicating complete spatial randomness. The statistical significance for the spatial autocorrelation relationship is determined by standardizing the statistic Z value [33]. At a confidence level of 0.05, |Z| = 1.96; at a confidence level of 0.01, |Z| = 2.58. The significant or highly significant level was set when |Z| > 1.96 or |Z| > 2.58.

Second, Anselin Local Moran’s I statistic was applied to examine disease spatial autocorrelation at the local level. Unlike the global Moran’s I, the expected value of local Moran’s I varies for each sampling village because it is calculated in relation to its particular set of neighbours [34]. The local Moran’s I index identified locations of clusters or hotspots where the value of the index was extremely pronounced across localities, as well as those of spatial outliers [35]. The significance of the local Moran’s I was calculated using a randomization test on the Z-score value [36]. A positive Z-score value indicates that the health loss of KBD in one village is surrounded by similar health loss in neighboring locations (high-high or low-low), thus forming a spatial cluster. A negative Z-score value indicates that the high health loss of KBD in one village is surrounded by low neighbors (high-low) and vice versa (low-high). Similarly, the significance level was set when |Z| > 1.96. |Z| ≤ 1.96 indicates presence of a random distribution. The results were mapped to display the specific locations of clusters (high-high and low-low) and potential outliers (high-low and low-high). Both the global and local spatial autocorrelation analysis were conducted in ArcGIS 10.5 (Esri, Redland, CA, USA) using spatial statistics tools.

### Statistical analysis

Data processing and chart production were mainly done using SPSS 26.0 (IBM Corp., Armonk, NY, USA), ArcGIS 10.5, and OriginPro 8.0 (OriginLab Corp., Northampton, MA 01060 USA). Spearman’s correlation coefficients for YLDs, YLD rate, prevalence and environmental factors were calculated. The differences of YLDs among ages, genders and various KBD grades were tested using a two-tailed Student’s t*-*test at 0.05 significance.

## Results

### Study population

Overall, 1.34% of the investigated population were reported suffering from KBD in Bin County, with no new cases discovered in children. The age of the patients ranged from 19 to 97 years old. Amongst patients with KBD, 52.9% were males and 47.1% were females. Patients with KBD of grade I, II and III accounted for 57.4, 37.4 and 5.3% of the total amount, respectively. 44.6% of them had been taking medical treatment for a long-term. Farmers were the main occupation for KBD patients. 39.9% never received education, and 19.2% were from poor poverty-stricken households whose annual net income per capita was lower than 2950 RMB.

### Age-sex distribution of YLDs for KBD

Table 1 showed the calculated YLDs in different KBD grades by gender. The total health loss from KBD in Bin County in 2018 was estimated at 858.78 YLDs (2.89 YLD per 1000 population, 53.8% for males and 46.2% for females). YLDs for males were higher in all grades of KBD than those for females, but with no statistical significance (*p* > 0.05). Among different KBD grades, KBD of grade II contributed most to the YLDs, followed by KBD of grade I, accounting for 54.4 and 31.0% of the total YLDs respectively. The same trends were observed for males and females. When compared with the prevalence rate of KBD, it was found that there was no consistent corresponding relationship. Although the largest contribution of YLDs was KBD of grade II (1.58 YLD per 1000 population), the highest prevalence rate was observed in KBD of grade I (0.77%).

**Table 1:** Years lived with disability (YLDs) for KBD in Bin County (2018)

KBD grades	Male	Female	Total		Prevalence (%)
	YLDs	YLDs	YLDs	YLD/1000	
I	136.42	129.87	266.29	0.90	0.77
II	258.99	208.59	467.58	1.58	0.50
III	66.82	58.10	124.92	0.42	0.07
Total	462.23	396.56	858.78	2.89	1.34

**Fig. 2:**
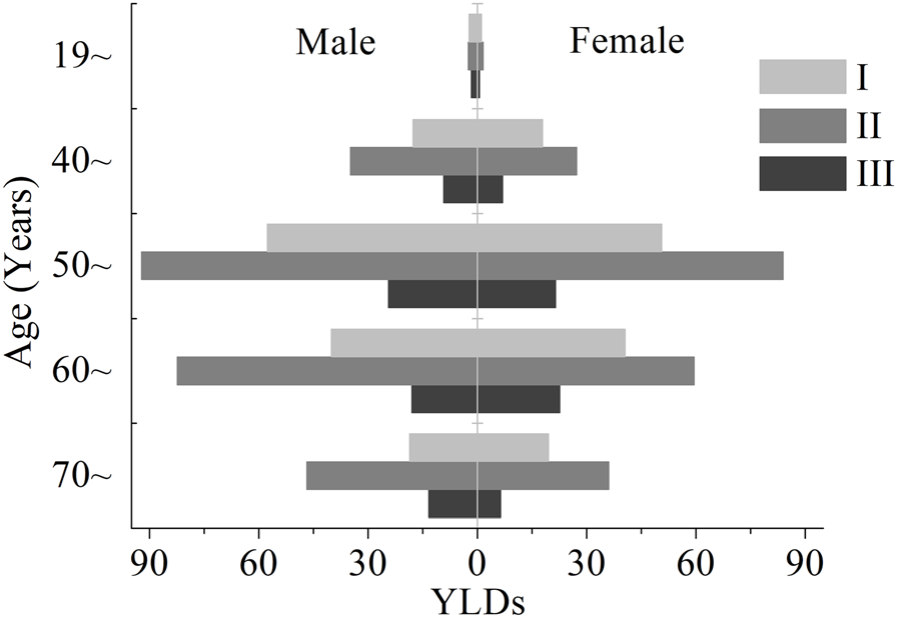
Age-sex specific YLDs among KBD grades in Bin County

Figure 2 displayed the age-specific distribution of YLDs between genders and KBD grades in Bin County. According to the distribution of the number of patients, age was divided into five groups: 19~, 40~, 50~, 60~, 70 years and over. The age group of 0 ~ 18 was not included in Fig. 2 as there were no cases under the age of 18 years. The results showed that YLDs increased with age and decreased at the highest 50 ~ age group (330.49 YLDs, 38.5% of total YLDs). Approximately 85.6% of the total YLDs were from the age group of 50 years and above. YLDs in KBD of grade II were the highest in all age groups, followed by KBD of grade I. There were no significant differences in YLDs among KBD grades (*p* > 0.05). These trends were generally consistent in the two genders, only with relatively high YLDs for males in all age groups.

### Spatial distribution of health loss from KBD

**Fig. 3:**
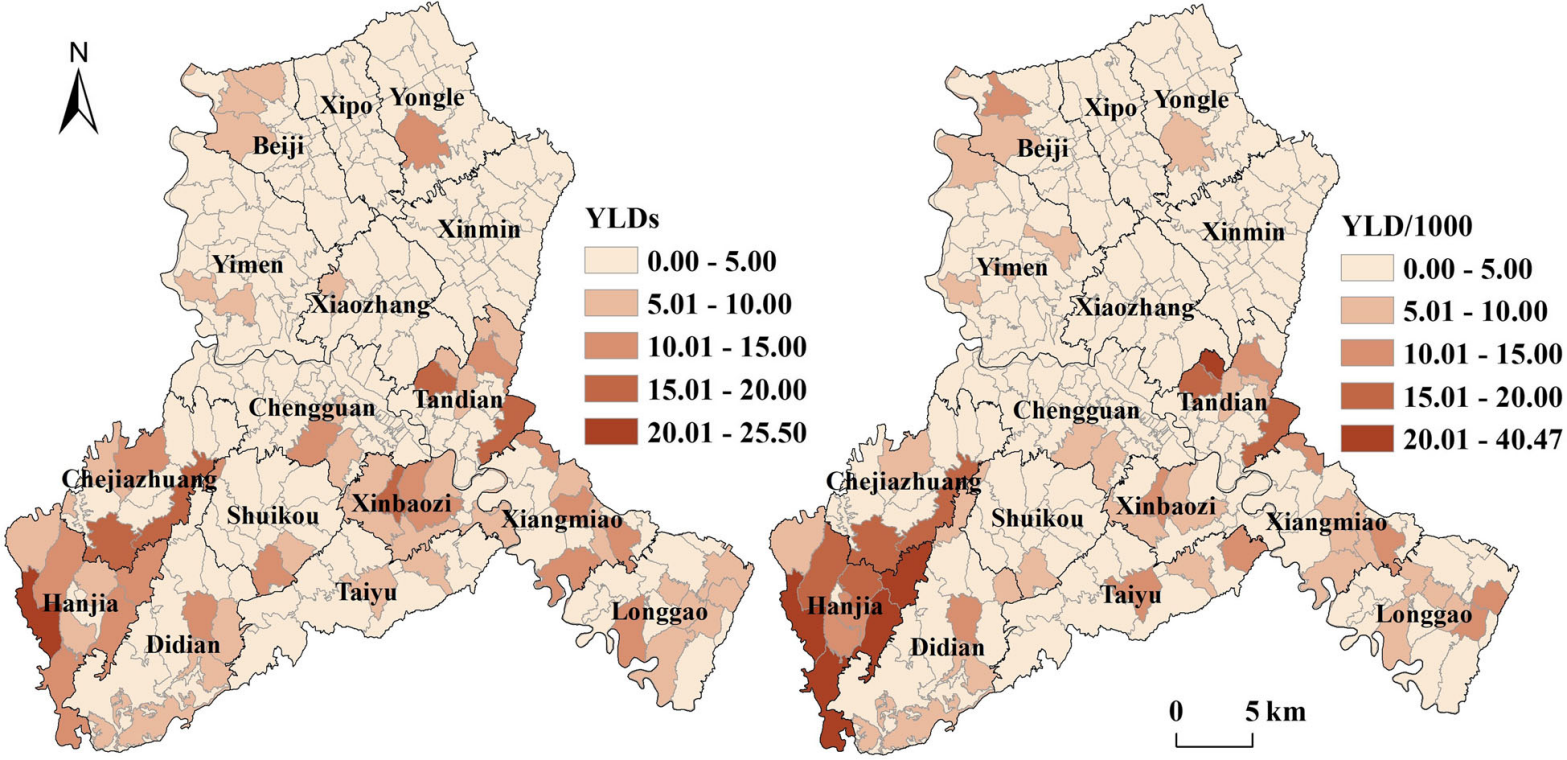
Spatial distribution of YLDs (left) and YLD rate (right) for KBD in Bin County

Figure 3 presented the spatial distribution of healthy life years loss from KBD in Bin County at the village-scale. Obvious regional variations were observed in both YLDs and YLD rate, with higher values in the southern regions and lower values in the northern regions of Bin County. Areas with the most serious health loss were mainly clustered in the southwest. The loss of healthy life years from KBD in villages of Bin County ranged from 0 to 25.50 YLDs, while their YLD rates had a larger variation, ranging from 0 to 40.47 YLD/1000. The top five townships with the highest YLD rates were Hanjia (19.83 YLD/1000, 97.16 YLDs), Xiangmiao (6.04 YLD/1000, 78.49 YLDs), Tandian (5.74 YLD/1000, 88.97 YLDs), Xinbaozi (5.54 YLD/1000, 73.43 YLDs), and Chejiazhuang (4.95 YLD/1000, 69.39 YLDs).

### Spatial autocorrelation and hotspot detection

To detect the spatial autocorrelations of YLDs and YLD rate, global Moran’s I statistics were calculated at village-level in Bin County. It was found that the Moran’s I index values for YLDs and YLD rate were 0.27 and 0.37, indicating that the distribution of health loss from KBD have positive correlations. The Z score, which is a standardized statistic, for YLDs and YLD rate were 7.607 (*p* < 0.0001) and 10.511 (*p* < 0.0001), respectively. Apparently, the global spatial autocorrelations of YLDs and YLD rate presented significant spatial clustering. Villages with KBD cases were not randomly distributed in space among all villages in Bin County. Similarly, the YLDs and YLD rate of different gender groups also showed significant spatial clustering at village-level. The Moran’s I index values of YLDs and YLD/1000 for males were 0.15 and 0.21 (*p* < 0.01), those for females were 0.12 and 0.17 (*p* < 0.01), respectively.

**Fig. 4:**
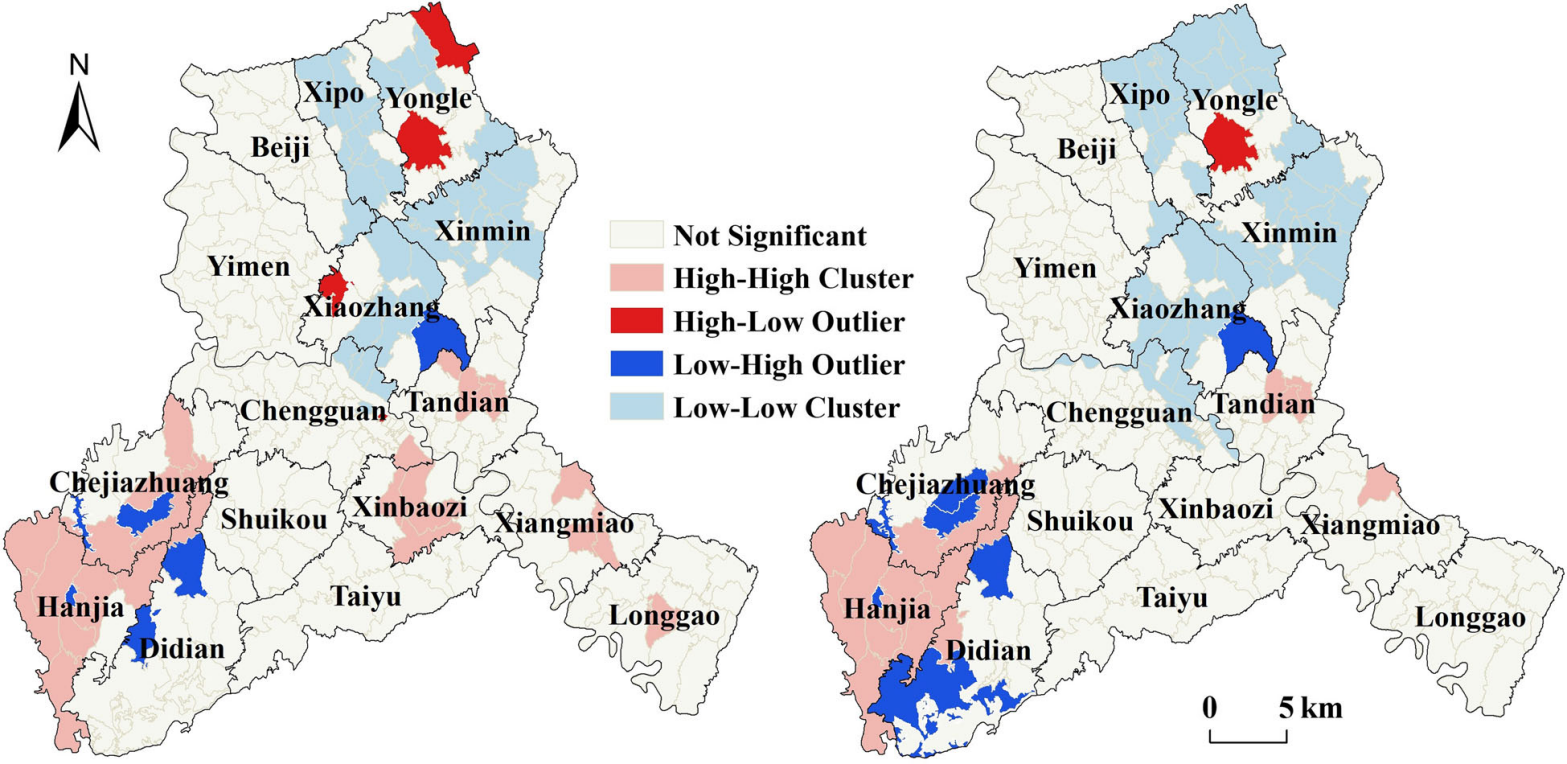
Spatial clusters of YLDs (left) and YLD/1000 (right) for KBD in Bin County

To further identify the locations of significant clusters (hot and cold spots), Anselin local Moran’s I index was applied at village-level in Bin County. Maps in Fig. 4 showed the locations with significant local Moran’s I statistics and classified those locations by type of association (LISA cluster map). The high-high and low-low clusters are indications of high values surrounded by high values and low values surrounded by low values. In contrast, the high-low and low-high locations are indications of spatial outliers. There were some outstanding spatial clusters of both YLDs and YLD/1000 observed in Bin County. The clustered villages with high YLDs and YLD/1000 (hotspots) were found to cover most areas of Hanjia and Chejiazhuang Townships in the southwest and parts of Tandian and Xiangmiao Townships in the east of Bin County. The clustered villages with low YLDs and YLD/1000 (cold spots) were mainly found in the north and central of Bin County, covering Xipo, Yongle, Xinmin, Xiaozhang and Chengguan Townships. The hotspots of YLDs were slightly different from that of YLD/1000. Several clustered villages with high YLDs were also observed in Xinbaozi and Longgao Townships, discontinuously distributed in the southeast of Bin County.

### Natural and social determinants of health loss

We computed Spearman’s correlation coefficients and its 95% confidence intervals to investigate relationships between the health loss and variables of interest (Table 2). Natural factors related to environmental Se contents and social factors including the poverty rate and educational attainment were selected for the correlation analysis. Environmental Se including the total Se in soil and local staple crops (i.e. wheat) as well as several major Se speciation in soil (i.e. available Se speciation and organically bound Se) were taken into consideration. The results showed that YLDs and YLD rate of KBD had no significant correlations with total Se contents in soil and wheat as well as soil available Se, but were positively and significantly correlated with organically bound Se in soil (*r*_*YLDs*_ = 0.216, *r*_YLD/1000_ = 0.217, *p* < 0.05, *N* = 83). By contrast, the prevalence of KBD had no significant correlations with any environmental Se variables. As for social factors, only the poverty rate of patients with KBD showed significantly positive correlations with YLD rate (*r* = 0.267, *p* < 0.05, *N* = 75) and prevalence rate (*r* = 0.264, *p* < 0.05, *N* = 75).

**Table 2:** Spearman’s correlation coefficients for YLDs, YLD rate, prevalence and environmental factors

	Natural environmental factors				Socio-economic factors	
	Soil Se	Wheat Se	Available Se^a^	Organic Se	Poverty	Education
YLDs	0.153	−0.008	−0.052	0.216^b^	0.203	0.223
YLD/1000	0.150	0.064	−0.070	0.217^b^	0.267^b^	0.220
Prevalence	0.140	0.089	−0.060	0.213	0.264^b^	0.213

^a^Available Se represents water-soluble and exchangeable fractions of Se in soil

^b^indicates significant (*p* < 0.05)

## Discussion

One of the relevant contributions of using YLDs is that they give disability weights in different degrees according to the severity of KBD, while simultaneously taking its prevalence and severity into account. Consequently, the ranking of the health loss caused by KBD differs from the ranking based on prevalence rates (Table 1). Similar findings can be found in many other relevant studies [10, 11, 37, 38]. Compared with traditional index (e.g. prevalence), YLDs is more conductive to the measurement of these non-fatal consequences and the exploration of environmental etiology of KBD.

This study showed that spatial distribution patterns of YLDs and YLD rate of KBD were significantly clustered, and identified their hotspots and cold spots in Bin County. Factors that might affect this spatial clustering were analyzed from natural and social environment. In the aspect of natural factors, the results showed that organically bound Se in soil significantly and positively affected the distribution of YLDs and YLD rate. Organically bound Se is known as the unavailable fraction in soil, which is mainly found in the fulvic acid and humic acid of soil humus [26]. Previous studies have revealed that the organic matter (mainly fulvic acid) in drinking water may be an etiological factor of KBD [39]. The total amount of organic matter and humic acid in drinking water from KBD endemic areas were significantly higher than those in non-endemic areas, and changes in water sources effectively prevented the occurrence of KBD [40, 41]. Bin County is located in the southwest of Loess Plateau, where soil erosion is very serious [42]. Organic compounds in soil such as humic acid can easily leach into water under such circumstance, consequently bringing about threats to human health. Therefore, the health loss of KBD may be directly associated with the distribution of organic matters in soil. The non-significant relationship between prevalence rate and organically bound Se is possibly due to the limitation and one-sidedness of this index, which only counts the total number of cases and fails to reflect the harm and severity of different KBD grades comprehensively. With regard to total Se contents in soil and wheat, their non-significant correlations with both YLD rate and prevalence rate indicate that the dependence of local residents on low-Se environment may be weakening. With the improvement of living standards and measures of Se supplement, the food sources of Se intake increase. Residents do not have to entirely rely on their local crops.

In aspect of social factors, positive and significant correlations were observed for YLD rate and prevalence with poverty rate. This is consistent with previous findings of the impact of economic levels on life quality of patients with KBD [23]. KBD tends to occur in remote areas with weak infrastructure and inconvenient transportation. These regions are usually poverty-stricken areas. Poor economic conditions restrict the improvement of living and nutritional levels of local residents, thus increasing the risk of the occurrence and development of KBD. Some studies even directly attributed the level of economic income as the main factor affecting the occurrence of KBD [43, 44]. Moreover, positive but non-significant correlations were observed for the indexes with educational attainment, indicating that people with higher education levels suffered more health loss. This result does not accord with previous findings. According to Chen et al. [22], those with higher education levels tended to easily acquire and accept health knowledge of KBD, thus early diagnosing and treating the disease. By comparison, the positive correlation of this study may be attributed to the generally poor education attainment of patients in Bin County. Among the educated population, 72.0% received primary education only, which may not be substantively helpful with the prevention of KBD.

Some limitations should also be noted. First, reliable sources of disability weight are required to calculate YLDs. Disability weights of different sequela of KBD in the present study were based on the results of rheumatoid arthritis in GBD 2017. It was assumed that these data would be acceptable as the two diseases have similar symptoms according to the health state lay descriptions in GBD 2017 disability weights dataset [31]. Second, discounting and age weighting were not considered when calculating YLDs. Given the long duration, non-fatal outcomes and late-onset serious symptoms of KBD, which is quite similar to other endemic diseases such as schistosomiasis and goiter, we adopted the same strategy carried out in the GBD study of these two diseases [7, 45]. Namely, the duration of the disease was hypothesized as 1 year. Consequently, there is no discounting problem. With regard to age weights, the original GBD study weighted a healthy life lived at very young and old ages lower than other ages [46]. However, this is still controversial, since not all such studies agree that the youngest and oldest ages should be given less weight, nor do they agree on the relative magnitude of the differences [10, 46, 47]. Thus, the social values were not considered in this study. Third, given that the income variable of each patient or family is not available in the dataset, we instead used the poverty rate of each village, which failed to distinguish the effect of specific economic levels. Finally, the effect of population age structure on the distribution of health loss of KBD was not considered in the analysis mainly due to the insufficiency of these data in the study area. It should be noted that 85.6% of the total YLDs in Bin County were from the age group of 50 years and above (Fig. 2), indicating that a higher proportion of aging population may lead to a greater loss of healthy life years.

## Conclusions

The present study found that the health loss from KBD in Bin County was significantly clustered in spatial distribution. This clustering was collectively affected by environmental Se and socio-economic factors. In particular, organically bound Se in soil and poverty rate might be the most influential natural and social factors. Our results suggest that future treatment and prevention of KBD should focus on endemic areas with high organically bound Se in soil and poor economic conditions. These findings can be helpful for public health officials to formulate targeted policies and also provide important information for further exploration of the environmental etiology of KBD.

## Supplementary Information

**Additional file 1.**

## Data Availability

The datasets collected and analyzed during the current study are not publicly available due to confidentiality requirements, but are available from the corresponding author on reasonable request and with permission of the Binxian Center for Disease Prevention and Control.

## Abbreviations

KBD: Kashin-Beck disease
DALY: Disability adjusted life year
YLDs: Years lived with disability
