# Supplementary material for: Spatial distribution and determinants of health loss from Kashin-Beck disease in Bin County, Shaanxi Province, China
Source: BMC Public Health. 2021 Feb 19;21:387. doi: 10.1186/s12889-021-10407-6 (PMC7893884; doi:10.1186/s12889-021-10407-6)
Supplement: Supplementary file 1 — Additional file 1. [file 12889_2021_10407_MOESM1_ESM.docx]

**Spatial distribution and determinants of health loss from Kashin-Beck disease in Bin County, Shaanxi Province, China**

Jing Wang^1,2^, Xiaoya Wang^2^, Hairong Li^2,3^*, Linsheng Yang^2,3^*, Yingchun Li^4^, Chang Kong^2^

^1^ Key Laboratory for Geographical Process Analysis & Simulation, Research Institute of Sustainable Development, Central China Normal University, Wuhan 430079, China

^2^ Key Laboratory of Land Surface Pattern and Simulation, Institute of Geographical Sciences and Natural Resources Research, Chinese Academy of Sciences, Beijing 100101, China

^3^ College of Resources and Environment, University of Chinese Academy of Sciences, Beijing 100049, People’s Republic of China

^4^ Binxian Center for Disease Prevention and Control, Binxian 713500, China

*Corresponding author: Hairong Li, Linsheng Yang

Email: [lihr@igsnrr.ac.cn](mailto:lihr@igsnrr.ac.cn); [yangls@igsnrr.ac.cn](mailto:yangls@igsnrr.ac.cn)

**Interview guide for Kashin-Beck disease (KBD) patients**

1. Address: Township Village

2. Name 3. Gender

4. Age 5. Ethnic group

6. Occupation 7. Educational attainment

8. Poverty-stricken family Yes 🞎 No 🞏

9. Disability Yes 🞎 No 🞏

If yes, the degree of disability is

10. Clinical diagnosis of KBD I 🞎 II 🞎 III 🞎

11. Staple food Rice 🞎 Wheat 🞎

12. Source of staple food Self-produced 🞎 Purchased 🞎
